# Understanding the sequence requirements of protein families: insights from the BioVis 2013 contests

**DOI:** 10.1186/1753-6561-8-S2-S1

**Published:** 2014-08-28

**Authors:** William C Ray, R Wolfgang Rumpf, Brandon Sullivan, Nicholas Callahan, Thomas Magliery, Raghu Machiraju, Bang Wong, Martin Krzywinski, Christopher W Bartlett

**Affiliations:** 1 Nationwide Children's Hospital, 575 Children's Crossroad, 43215, Columbus, OH, USA; 2 The Ohio State University, 100 W. 18th Ave, 43210, Columbus, OH, USA; 3 The Broad Institute, 7 Cambridge Center, 02142, Cambridge, MA, USA; 4 Genome Sciences Centre, 570 W, 7th Avenue, V5Z 4S6, Vancouver, BC, Canada; 5 Contest Chairs; 6 Domain Experts

## Abstract

**Introduction:**

In 2011, the BioVis symposium of the IEEE VisWeek conferences inaugurated a new variety of data analysis contest. Aimed at fostering collaborations between computational scientists and biologists, the BioVis contest provided real data from biological domains with emerging visualization needs, in the hope that novel approaches would result in powerful new tools for the community. In 2011 and 2012 the theme of these contests was expression Quantitative Trait Locus analysis, within and across tissues respectively. In 2013 the topic was updated to protein sequence and mutation visualization.

**Methods:**

The contest was framed in the context of a real protein with numerous mutations that had lost function, and the question posed "what minimal set of changes would you propose to rescue function, or how could you support a biologist attempting to answer that question?". The data was grounded in actual experimental results in triosephosphate isomerase(TIM) enzymes. Seven teams composed of 36 individuals submitted entries with proposed solutions and approaches to the challenge. Their contributions ranged from careful analysis of the visualization and analytical requirements for the problem through integration of existing tools for analyzing the context and consequences of protein mutations, to completely new tools addressing the problem.

**Results:**

Judges found valuable and novel contributions in each of the entries, including interesting ways to hierarchicalize the protein into domains of informational interaction, tools for simultaneously understanding both sequential and spatial order, and approaches for conveying some types of inter-residue dependencies. In this manuscript we document the problem presented to the contestants, summarize the biological contributions of their entries, and suggest opportunities that this work has highlighted for even more improved tools in the future.

## Introduction

The purpose of the BioVis contests are to engage the Visualization research community in developing novel solutions for, and new collaborations with the Bio/Life sciences, that address real, challenging problems in need of meaningful solutions. To accomplish this, the BioVis symposium provides two contest tracks: 1) a Data Analysis track that seeks to identify novel visualization and analytical approaches to a meaningful biological problem, by challenging entrants to solve it with combined visualization and bioinformatic approaches; and 2) a Redesign track that provides a single figure that fails to adequately communicate the intended content, and asks contestants to propose improved designs.

In 2013, the subject of both the Data Analysis, and Redesign contests was visualization related to protein sequence data. In the case of the Data Analysis contest, the challenge revolved around protein sequence-function representations, and took the form of a sequence for a mutated triosephosphate isomerase(TIM) protein that had acquired multiple point changes until it lost function (defective TIM - dTIM). The contest was posed in the form of identifying, or producing tools to identify, minimal sets of rescue mutations that might restore function to dTIM. Unlike a conceptual "toy" contest or a simulation, the dTIM data is from an actual experimental protein, produced in the lab and assayed for function. Likewise known multiple point rescue mutants have been produced and validated[1]. The Redesign contest posed a related problem in the form of a figure that attempted to show the difference in sequence requirements for two subfamilies of Adenylate Kinase(ADK). These two subfamilies produce a folded domain with essentially identical conformation, using two dramatically different biophysical strategies to stabilize the structure.

The Data Analysis contest was therefore fundamentally about understanding the consequences of the assorted mutations in dTIM. Making this challenge more interesting, none of the mutations that produced the functionally crippled phenotype, are outside the consensus sequence for TIM family proteins. In fact, all of the mutations in dTIM, are mutations *towards *the family consensus. While the crippled functionality of dTIM appears to fly in the face of conventional wisdom that most towards-consensus mutants are inconsequential, or increase protein stability and function, the crippled phenotype is really an example of the reality that some towards-consensus mutants are damaging to the protein. This occurs because not all amino acids in a protein contribute independently to its function. Some residues contribute in combination with other residues, and mutations towards the consensus can violate these interdependent contributions. As a result, while it is trivially easy to propose rescue mutations for dTIM that are reversions of the existing mutations toward a functional parent, it is entirely possible to rescue dTIM by mutating *other *residues to new amino acid identities. This occurs because all of the residue choices in dTIM are valid residues for those positions in some members, often the majority, of the TIM family,. Rescuing functionality requires only restoring functionally-required residue-dependency networks, and these can be restored without resorting to reversion of any existing dTIM mutations. This phenomenon is not widely discussed in the literature, because almost all current mutation-analysis approaches focus on single-point effects, and there is a dearth of tools for analyzing and understanding mutational networks. It was in the hope of inspiring new tools for such analyses, that the Data Analysis topic was chosen.

Where the Data Analysis contest was about understanding the defects in a single protein compared to its functional related family (where some of the defects were omission of required interdependencies in the family), the Redesign contest, was about understanding the differences between two (identically) functional families of proteins with evolutionarily and biophysically divergent histories. Like the Data Analysis contest, the differences had both positional and network characteristics. The canonical representation for family characteristics today, is a Sequence Logo[2] presentation of the per-position distribution of residue identities and (assuming independence) information distribution per position across the sequence. For the adenylate kinase domain presented (the lid domain, PFAM family ADK lid[3]), ADK has evolved two distinctly different ways of stabilizing the same structure. As a result, the identities in many positions are not independent, and Sequence Logos fail to convey the meaningful family characteristic of dependencies[4], as well as suffering from significantly sub-optimal presentation issues for comparing multiple families[5,6]. It was in the hope of identifying improved representations, that both captured important family features such as dependencies, and improved utility for comparative analysis, that the ADK lid domain families, represented as Sequence Logos, was chosen for the Redesign contest.

## Methods

For both 2013 BioVis contest tracks, the contest was announced, with data available immediately for download, at the 2012 BioVis contest on Oct. 15th 2012. A 45- minute primer was presented during the main conference schedule that highlighted the biological issues, cast them in terms familiar to the Visualization community audience, and outlined several opportunities and avenues for attack via different sub-domains of the visualization sciences. This presentation was followed by a 2.5- hour break-out session for interested parties where domain experts explained the data in more detail, answered questions from the attendees, and helped potential contestants identify connections between their work and contributions to the contest topics.

In addition, descriptions of the data, insights into the domain issues, and suggested approaches and tasks to focus upon were posted on the BioVis website (http://www.biovis.net/year/2013/info/contest and http://www.biovis.net/year/2013/info/redesign-contest for the Data Analysis and Redesign contests respectively).

A web-based discussion forum was available for, and used by contestants for publicly discussing questions and ideas, as well as by the domain experts to provide full exposure of answers to the entire contestant community. Several additional talks and primers were given at other related conferences such as VizBi 2013 (http://vizbi.org/), and announcements about the contest were shared with registered contestants through email, as well as with both the computational and biological communities through several dedicated-topic email lists.

### Data analysis contest

For the Data Analysis contest, the provided data consisted of: The sequence of a functionally defective triosephosphate isomerase mutant (dTIM) (248 residues), the *S. cerevisiae *triosephosphate isomerase (scTIM) parent of dTIM (also 248 residues), the 3D structure of scTIM (to which dTIM appears similar), full sequences of all other known TIMs (1.4Mb of sequences as defined by the PFAM TIM database), and a hand-curated sequence alignment corresponding to the 3D structural core core of the TIM family members considered to be the most relevant by the data providers(TM,BS), and from which dTIM was designed by Sullivan et al.[1]). The PDB structure of scTIM (PDB ID 1YPI) provided 3-dimensional coordinates of a protein with high sequence and validated structural similarity to dTIM, while Sullivan's hand-curated alignment provided for registering dTIM, and other TIM sequences or structures that the contestants chose to use, with the scTIM sequence and structure.

#### Triosephosphate isomerase

TIM is a 54-kD enzyme that catalyzes the reversible interconversion of the triosephosphate isomers dihydroxyacetone phosphate and D-glyceraldehyde 3-phosphate, and as such plays a vital role in the glycolytic pathway. Because of this fundamental role, TIM is moderately well conserved throughout a wide range of eukaryotes including humans, drosophila, yeast, and *C. elegans*. Structurally, TIM is a homodimer comprised of two 250-residue units. Each unit consists of 8 outer alpha helices with 8 parallel inner B strands, which together comprise the canonical "TIM barrel", with the active site of the enzyme located within the barrel.

Initially dTIM was developed as a towards-consensus variant of TIM based on the PFAM alignment of 639 TIM sequences. Despite representing the canonical consensus, dTIM showed significantly less activity than wild-type, and surprisingly had a monomer structure, rather than the expected dimer. Further work demonstrated that the functional deficit was not due to alterations of the active site itself, or due to ablation of required active-site geometry due to loss of dimerization. A rescue mutant, ccTIM was developed based on a more stringent TIM subfamily alignment. While ccTIM is more distant from its nearest homolog than dTIM, it shows a markedly greater activity[1].

#### Presentation to the contestants

**Figure 1:**
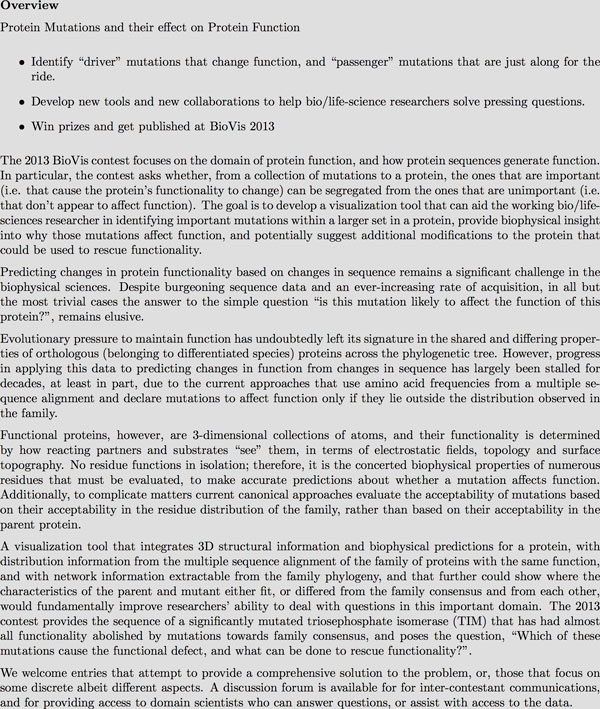
**Presentation of the Data Analysis contest problem on the BioVis website**.

The Data Analysis contest was presented to the contestants on the http://biovis.net/ website as shown in Figure 1.

**Figure 2:**
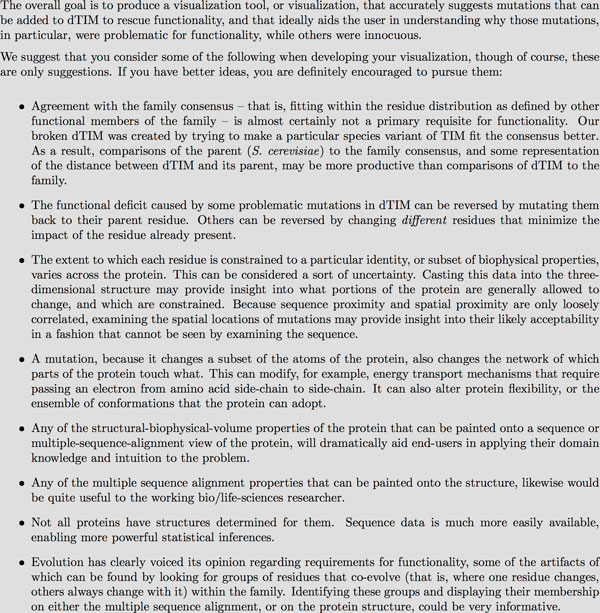
**Suggested approaches and topics that were given for entrants to focus upon in the Data Analysis contest**.

The website, as well as live presentations, emails and other communications regarding the contest, suggested that the contestants focus on certain topics as shown in Figure 2.

Submissions that addressed any of the suggested topics, as well as other ideas about visualizing the data, were accepted in the form of a 4 to 6-page written "answer", and an accompanying presentation, demo, or movie showing the approach in use.

#### Features of meaningful note and evaluation criteria

The triosephosphate isomerase dTIM mutant makes an ideal substrate for evaluating approaches to understanding the consequences of protein mutation. dTIM was constructed as a consensus mutant. As a result, every point mutation in it would be predicted, by existing mutation evaluation systems that focus exclusively on per-residue conservation and similarity to the overall family such as Mutation Assessor[7] and SIFT[8], to have no effect, or to actually improve dTIM's stability or function. Experimental results for dTIM (published as cTIM[1]), demonstrate that this is not the case. Additional results [9] indicate that there are multiple different ways that dTIM's functionality can be rescued, and all depend on identifying correlated networks of residues that are violated by the dTIM sequence.

These networks were only identified in the lab through a laborious and slow manual examination of the sequence characteristics and per-column-pair Joint Relative Entropy[10], coupled with extensive experimental validation. This process is far from ideal, and we are aware of only one analytical tool, StickWRLD, that can identify many of the important dependency-network features easily[11]. Unfortunately, StickWRLD does not couple its analysis to the biophysical and typical sequence properties of proteins in a way that facilitates use by a general protein-science researcher. Our hope in supplying dTIM as the target for this contest was that entrants would identify novel ways to identify and convey the important dependency network features, in the context of useful interfaces for protein data.

#### Judging

Entries were judged by two panels of three judges: A "Team Bio" group that included the domain experts most familiar with the specific TIM variant under study, who were tasked with evaluating whether the answers and approaches uncovered useful and/or novel insight into the system and the questions posed; and a "Team Vis" group that focused on whether the visualization approaches and design choices were appropriate, novel, and/or insightful. Each judging team chose a favorite entry amongst themselves, and then in collaboration both teams selected an overall favorite entry. Honorable mentions were also awarded to entries for specific features of interest or strengths called out by any of the judges.

The overall favorite entry was from Doncheva et al.[12], with an approach that melded several several canonical tools for protein sequence and structure analysis, with novel approaches for conveying folding and contact maps and the changes that mutations might make to these. Luciani et al.'s[13] entry was chosen as the Team Vis favorite for its thoughtful combination of features and for facilitating exploration of the data. Their entry also incorporated many common and well-accepted visualizations and tools for understanding the context of the mutations, and made significant use of 3D structural views as indices into, and summaries of, tabular mutation and similarity information. The entry from Silveira et al.[14] was selected as the Team Bio favorite, for producing the most successful tool in suggesting known-valid rescue mutations and other mutations with known stability-enhancing properties, as well as their good use of interface and representation paradigms familiar to the working biologist. Despite settling on these outstanding entries for awards, the rankings across the entire set of entries were very tight, and the judges found merit and novel value in every one of the entries submitted.

Additional details about each entry, from the judges and domain experts' perspective, are presented in the Results section below. Descriptions from the authors are included as separate manuscripts in this proceedings. Other than the placement of the three favorite entries at the front, ordering of the other articles here and in the proceedings are not indicative of judges evaluations or other measures of relative merit. Every entry presented in these proceedings in fact has unique strengths and outperformed all other entries for at least some analysis tasks.

### Redesign contest

**Figure 3:**
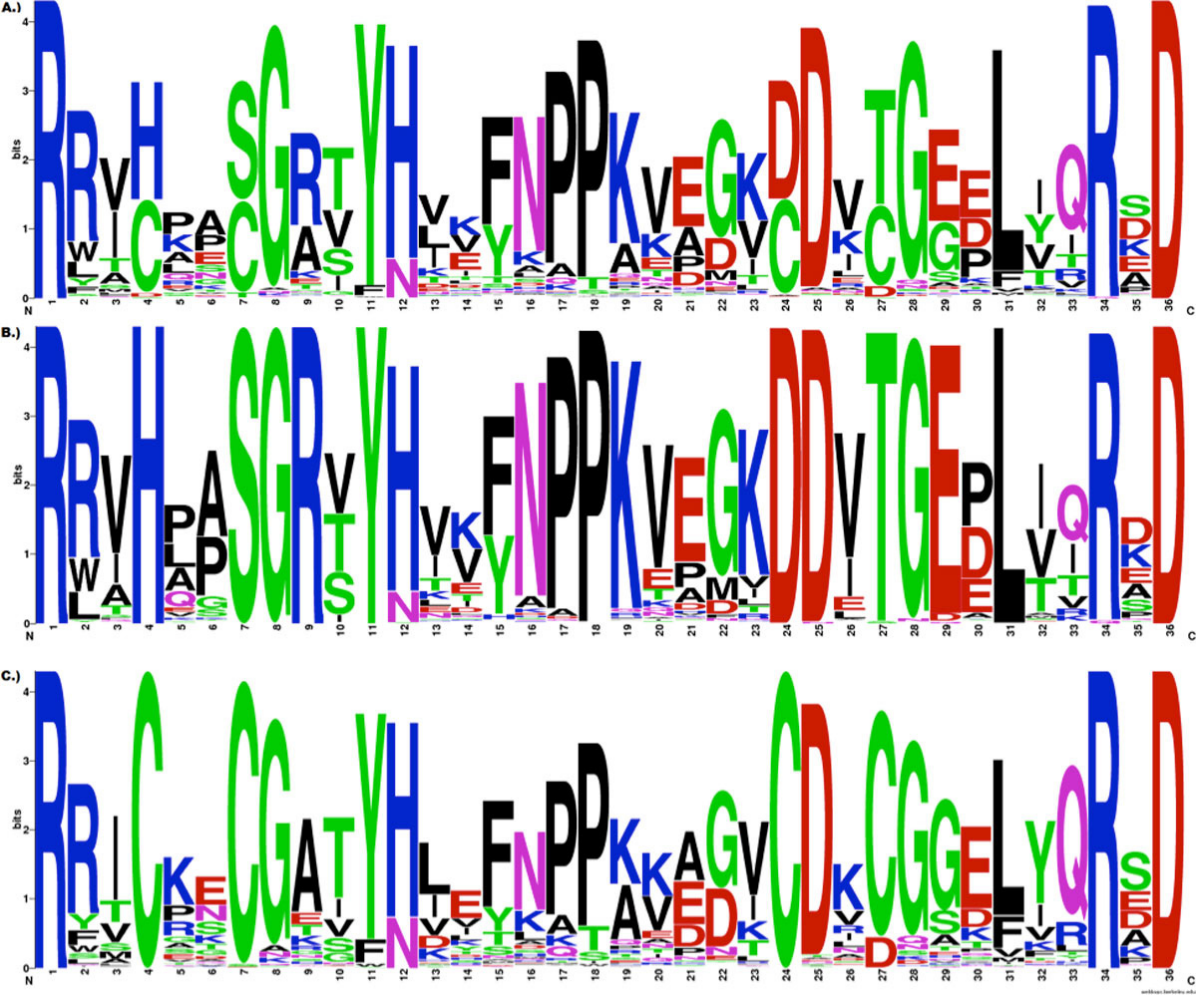
**The original ADK lid Sequence Logo figure for the Redesign Contest**. This image shows Sequence Logos for the ADK lid domain in (A) All organisms, (B) gram-negative bacteria, and (C) gram-positive bacteria, was used as the basis for the 2013 BioVis Redesign Contest.

For the Redesign contest, the data consisted of three Sequence Logo images (Figure 3), showing, respectively, the amino acid usage in the adenylate kinase lid domain (A) across all organisms, (B) from gram-negative bacteria and (C) from gram-positive bacteria, and also of the curated alignments used to generate these three images.

Sequence Logos are a frequently-used visualization tool for understanding certain properties of protein and nucleic-acid sequence families. They combine per-position information regarding which amino acids or nucleotides are the most prevalent in the family, with a theoretical evaluation of the amount of "information" about the visualized motif that is present at each position in the sequence. In simple terms, the (in-column) relative height of a given amino acid character in a position gives information about how frequently that amino acid appears in that position, while the relative heights of the columns give information about where in the motif, important features that make the family distinct from random sequences are located.

For the purpose of understanding the important features of sequence families where inter-positional dependencies are signature features, as we see in the ADK lid domain, Sequence Logos have significant limitations. Because Sequence Logos focus attention on information at single positions, information that is shared across multiple positions is invisible. As a result, it is entirely possible for the key residues in a sequence family, to display zero-height columns in a Sequence Logo visualization. Columns heights that significantly disguise the importance of a residue because that importance is shared, are common when visualizing protein families, and nearly ubiquitous in structural nucleic-acid motifs [15,4]. In the case of the ADK lid, there are significant dependencies between many positions, and the specific dependencies that are important, are conditional on the subfamily. The Sequence Logo rendition of these subfamilies does not make this information apparent to the viewer. There are also other representational issues that may yield to improved visualization ideas, such as the conflation of decreased visual salience for the "squashed" symbols that are infrequently represented in the family, and "squashed" symbols that are frequently represented, but in a supposedly low-information column.

In addition to the problems that make Sequence Logos a poor choice for the ADK lid data considered in the Redesign Contest, displaying multiple separate Sequence Logos, or any other visual representation, as a way of comparing different families, suffers from other presentation/understanding issues. The root of these issues lies in change and inattention blindness. Refocussing attention between two different figures to understand a change, results in decreased recognition of the change[5,6]. Used comparatively in this fashion, any visualization method that requires looking at multiple figures, limits the user to understanding only the details on which they focus their attention. Searching a pair of detailed representations of sequence families, such as the Sequence Logo visualization provided, requires the user to glance back and forth between the representations, individually identifying and noting each feature, its position and relationships to its neighbors, and then to look to the opposite figure and search out, and compare that mentally-recorded information to the details of the feature as present in the second visualization. This process is both laborious, and subject to significant errors due to its dependence on user memory for retaining and performing the comparison.

By presenting data that made these issues particularly problematic, with a short and otherwise easily described pair of sequence subfamilies, the Redesign contest aimed to identify improved sequence family representations that addressed both the in-family dependency features and between-family comparative needs that make data such as the ADK lid family problematic for Sequence Logo representations.

#### Adenylate kinase and its lid

ADK is a monomeric enzyme that catalyzes the breakdown of two molecules of ADP into one AMP and one ATP. This enzyme is seen in many species and is essential to metabolism in both prokaryotes and eukaryotes. ADK contains 3 essential domains - ATP-binding, AMP-binding, and the lid domain. Following substrate binding these domains fold over the active site in a coordinated fashion. The lid domain in particular shows distinct sequence, but not structural differences in gram-positive versus gram-negative bacteria. The ADK lid domain structure is universally conserved, but is stabilized in the gram-negatives by a hydrogen bonding network between residues 4, 7, 9, 24, 27, and 29 (and several other residues in some organisms), while the gram-positives are stabilized by a bound metal ion, tetrahedrally coordinated by the Cysteines at 4, 7, 24 and 27 (residue positions as per the PFAM ADK lid family). The identities of several other positions (e.g. 5, 8, 30, 32) are differentially constrained in each subfamily as well, apparently due to steric requirements of the stabilizing residues[4]. The most common way of representing sequence family characteristics, Sequence Logos, displays the properties of the overall adenylate kinase lid family, and of the subfamilies, as shown in Figure 3, however, these images are relatively uninformative about the actual features of the ADK subfamilies that make them unique.

#### Presentation to the contestants

**Figure 4:**
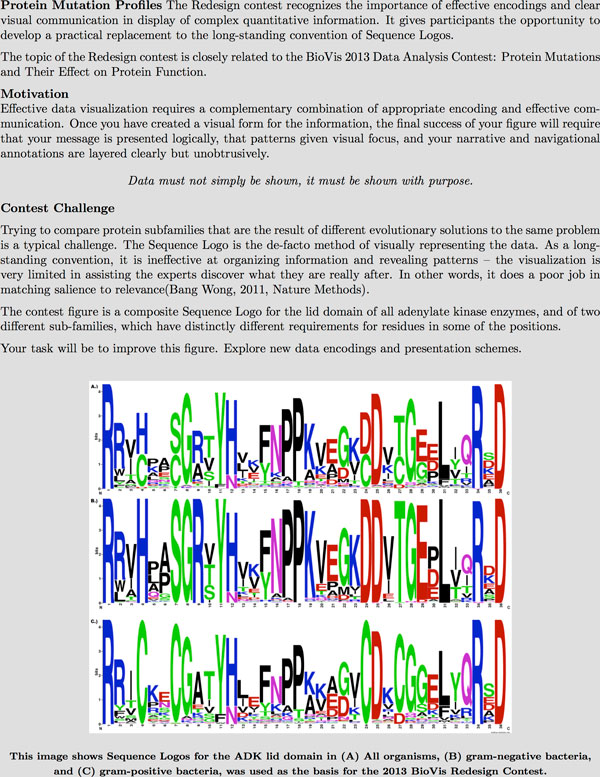
**Presentation of the Redesign contest problem on the BioVis website**.

The Redesign contest was presented to the contestants on the http://biovis.net/ website as shown in Figure 4.

**Figure 5:**
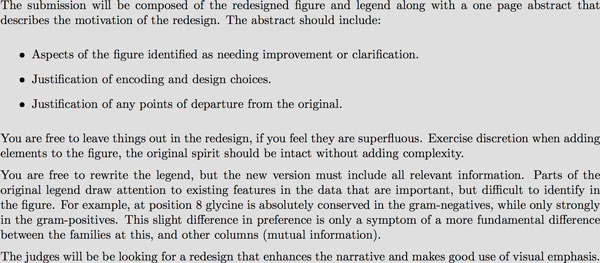
**Submission requirements and topics that contestants were encouraged to address for the Redesign contest**.

The contestants were instructed to create submissions as shown in Figure 5.

Submissions were accepted in Nature Publishing Group format (http://www.nature.com/nature/authors/gta/#a5.9).

#### Features of meaningful note and evaluation criteria

The adenylate kinase lid is a particularly interesting case for comparing sequence-family representations, because it contains several sequence features that make, for example, Sequence Logo representations, less than informative. There are networks of residues that have evolved cooperatively, but differently in two different branches of the phylogeny. There are mutually symmetric inter-residue dependencies where several residues must co-occur for ADK to be functional, and asymmetric dependencies where the presence of one residue identity implies a second, but the second does not imply the first. There are areas where evolutionarily homologous residues have shifted physical register. All of these features are meaningful characteristics of the family, and the differences between the ways that the gram-negative and gram-positive bacteria use these features, are important details for a useful figure representing the sub-families to convey.

We supplied the entrants with an explicit statement of the most important of these features (the differentially conserved networks in the gram-positives and gram-negatives around residues 4, 7, 9, 24, 27, and 29), as well as hints at other features of interest, in the hope that entrants would identify novel ways of showing both the per-position sequence characteristics in a more useful fashion, as well as incorporating visual features that highlight the importance of these residue dependency networks as primary signatures of this protein family.

Another important issue we hoped that the entrants would address, is change-blindess induced by needing to visually swap between two different figures for comparative analysis[5].

#### Judging

Entries were judged by the two Redesign Committee Chairs, based on a combination of the entry's intrinsic ability to convey the necessary information for its intended purpose, and the extent to which the entry capitalized on pre-existing visual semantics, thereby reducing the extent to which it would require explanation if substituted for a Sequence Logo figure in a manuscript.

The winning entry was from Heike Hofmann, with a fairly literal recapitulation of Sequence Logos in a comparative mode that interdigitates the residues from the multiple different sequence families being compared, into a single figure. Tied for second place were entries from Kultys et al.[17], and Sakai and Aerts[18]. Both of these entries applied a parallel-coordinates-like technique to represent the "trajectory" of each family member through its sequences' choices at each position.

Additional details about the winning entries, from the judges and domain experts' perspective, are presented in the Results section below. Descriptions from the authors are included as separate manuscripts in this proceedings.

## Results and discussion

### Data analysis

Doncheva et al.: overall favorite entry

**Figure 6:**
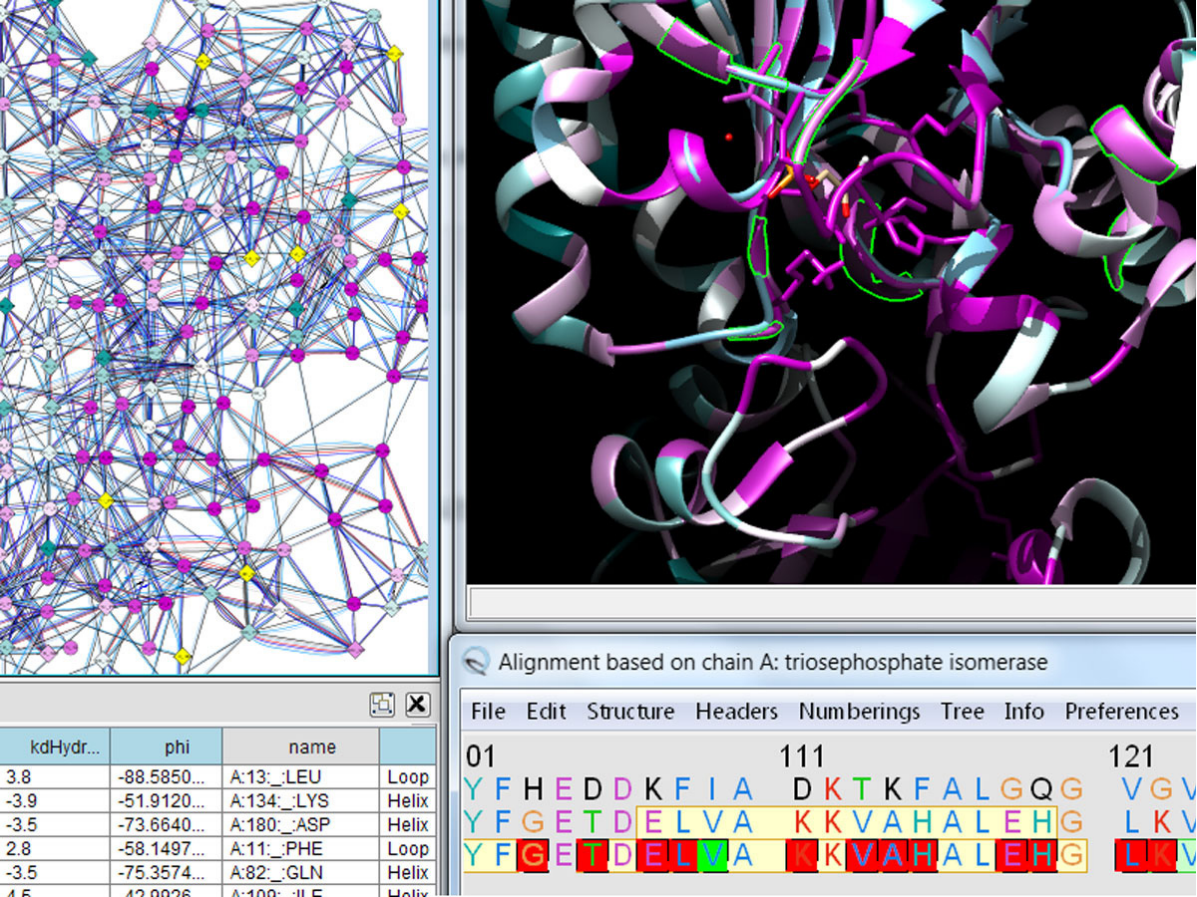
**RINalyzer in action**. RINalyzer displays both canonical sequence and structure information, as well as an eponymous Residue Interaction Map showing inter-residue contacts displayed by different family members and providing insight into the conservation of residue interaction.

Doncheva et al.[12] wrapped several traditional tools for understanding sequence and structure conservation, around the unique idea that changes in residue-residue contacts might explain functional deficits in mutant proteins. To support exploration of this phenomenon they developed the idea of a family Residue Interaction Network (RIN), which essentially visualizes structurally detectable residue-residue contacts in each member of a protein family for which there are known structures. From the RIN, shown interacting with other analysis features in Figure 6 it's easy to see where there are highly-conserved residue-contacts and networks of contacts in the family, enabling examination of the changes in a mutant to be considered in the context of how these changes might affect the highly conserved contacts. In addition to the Residue Interaction Network view, RINalyzer incorporates a sequence view, a map-like overview of secondary structural layout (Pro-Origami[19]), and a 3D molecular viewer, with feature selection synchronized between all of the views.

Their tool is very effective at providing the user several different perspectives of the protein family and mutant being compared, and has the ability to pull in additional information from external tools and databases. While the approach of integrating existing tools and displaying their output in individual panes may feel less "integrated" than a ground-up approach, it simplifies the incorporation of additional tools in the future.

Luciani et al. : Team vis favorite entry

**Figure 7:**
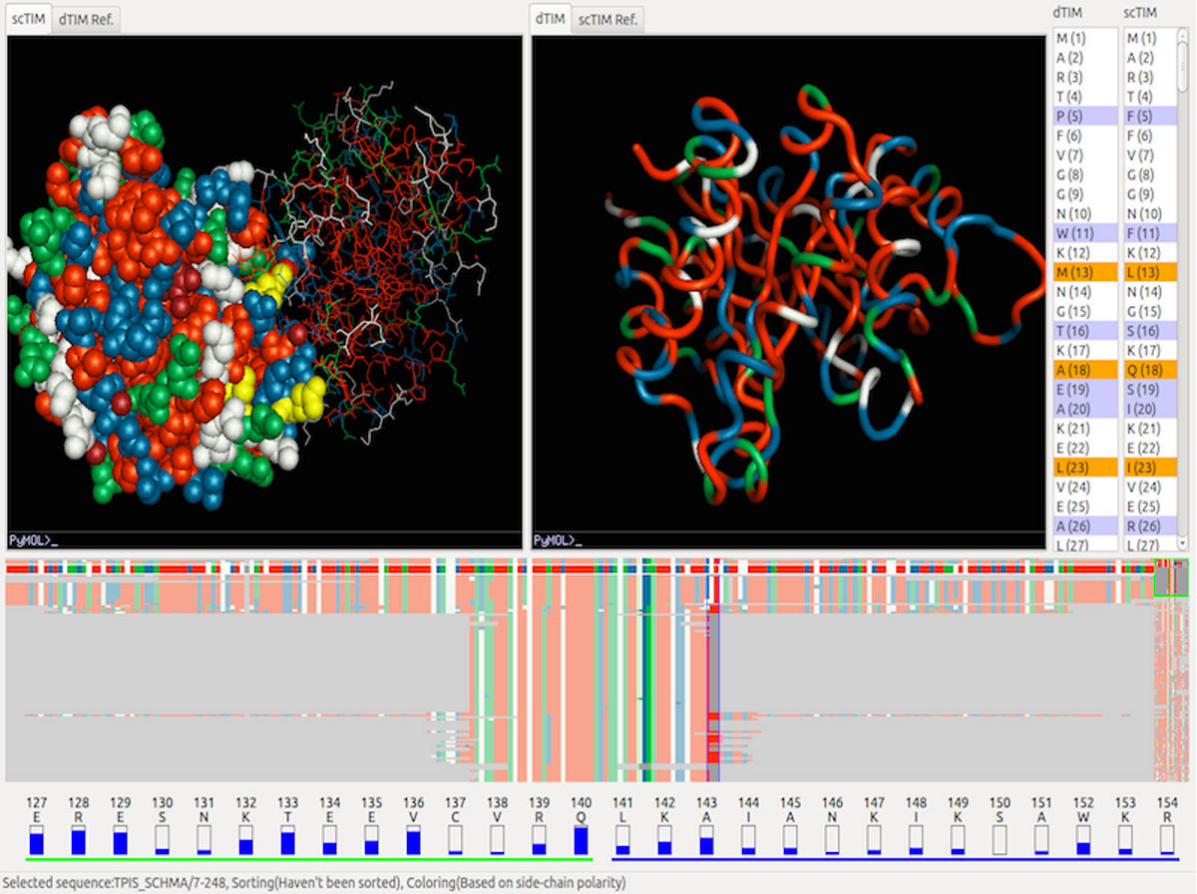
**Views from FixingTIM**. FixingTIM gives gives the user easy access to structural and sequence conservation properties of dTIM versus functional TIMs.

Luciani et al.[13] focused their approach to the problem around understanding mutations in the context of protein structure. The application, FixingTim, shown in Figure 7, successfully integrates multiple views of protein family data in one single application, merging and linking structure and sequence navigation. Instead of linking multiple independent windows with essentially independent but cooperating applications, FixingTIM brings multiple displays of different types of content along with the UI tools required to navigate them, into a single application with separate dedicated display panels.

The power of FixingTIM lies in the ability to pull in protein family sequence information and display the alignment, while simultaneously viewing the structure for any selected two members of the family. In this way, the user can compare the structure of interest against the structures of other members of the family, visually indicating the differences between them both in the interactive 3D view as well as in the sequence alignment view. The alignment view can be easily focused to specific regions, which dynamically updates a panel that displays the residue distribution within the family immediately below. FixingTIM makes the process of comparing two structures within the context of a protein family as easy and seamless as possible.

Silveira et al.: team bio favorite entry

**Figure 8:**
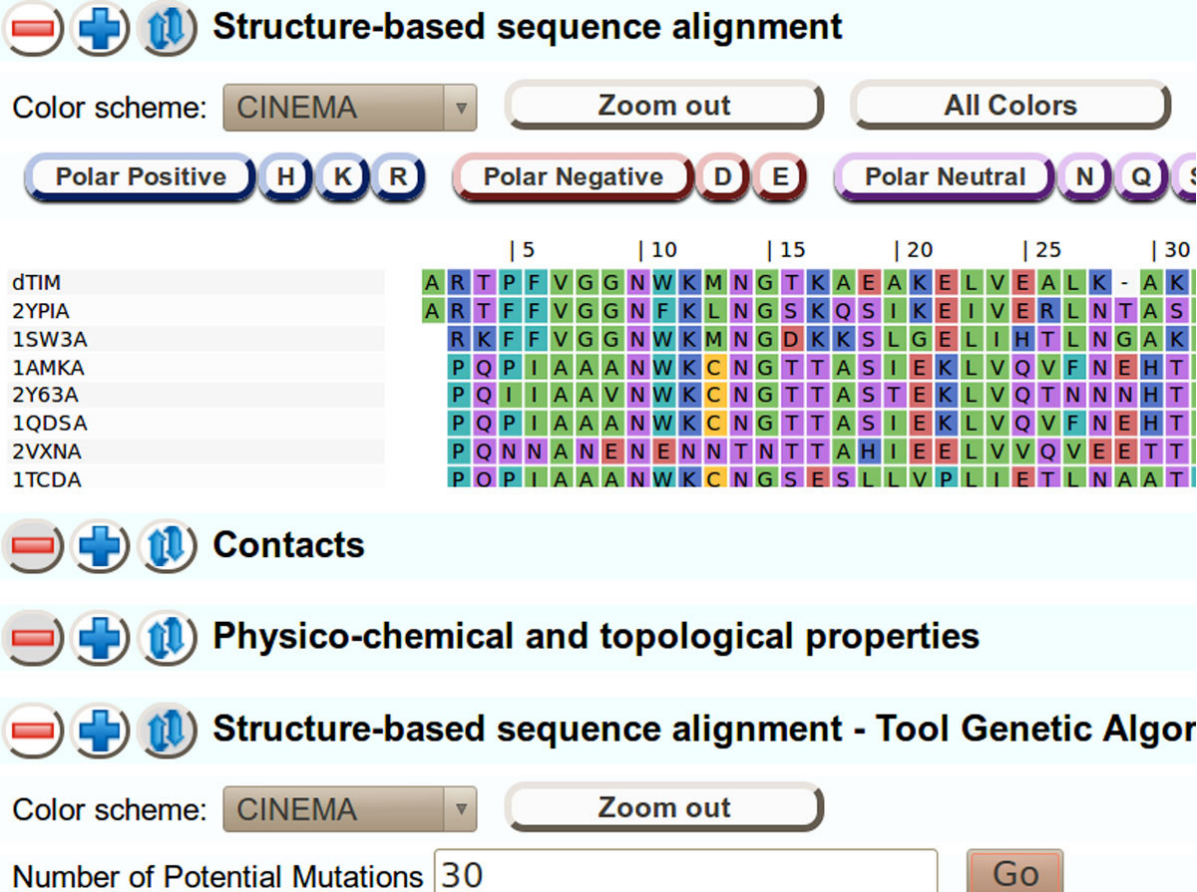
**A panel from VERMONT**. VERMONT compares dTIM to several functional TIMs, at the level of both residue identity and physicochemical similarity, guided by a trained expert system that focuses attention on potentially interesting features.

Silveira et al.[14] concentrated on appropriate selection and integration of diverse evidence about structural, and physicochemical consequences of mutations. Their tool, VERMONT, a panel from which is shown in Figure 8, is an interactive approach to exploration of protein sequences and structures viewed in terms of different types of conserved information. Similar to Doncheva et al.'s solution described previously, VERMONT integrates several existing packages - presenting the user with a variety of information with which they can make their own assessments. The modules include an alignment viewer, a panel for viewing residue contacts, and a physiochemical/topological properties panel.

Each of the individual views is fairly traditional on its own. What makes VERMONT a powerful tool is the integration of the pieces, and the presence of an automatic highlighting feature that uses a genetic algorithm to select and focus attention on residues and mutations that are most likely to have an effect on function. The approach is based on identification of mutations that deviate from the family norm in various physicochemical ways, and calculates a fitness for each based on a predetermined set of criteria. Residues that survive the genetic algorithm's "tournament" and are highlighted in the interface as hypotheses of interest with respect to functional impacts.

**Mercer et al**.

**Figure 9:**
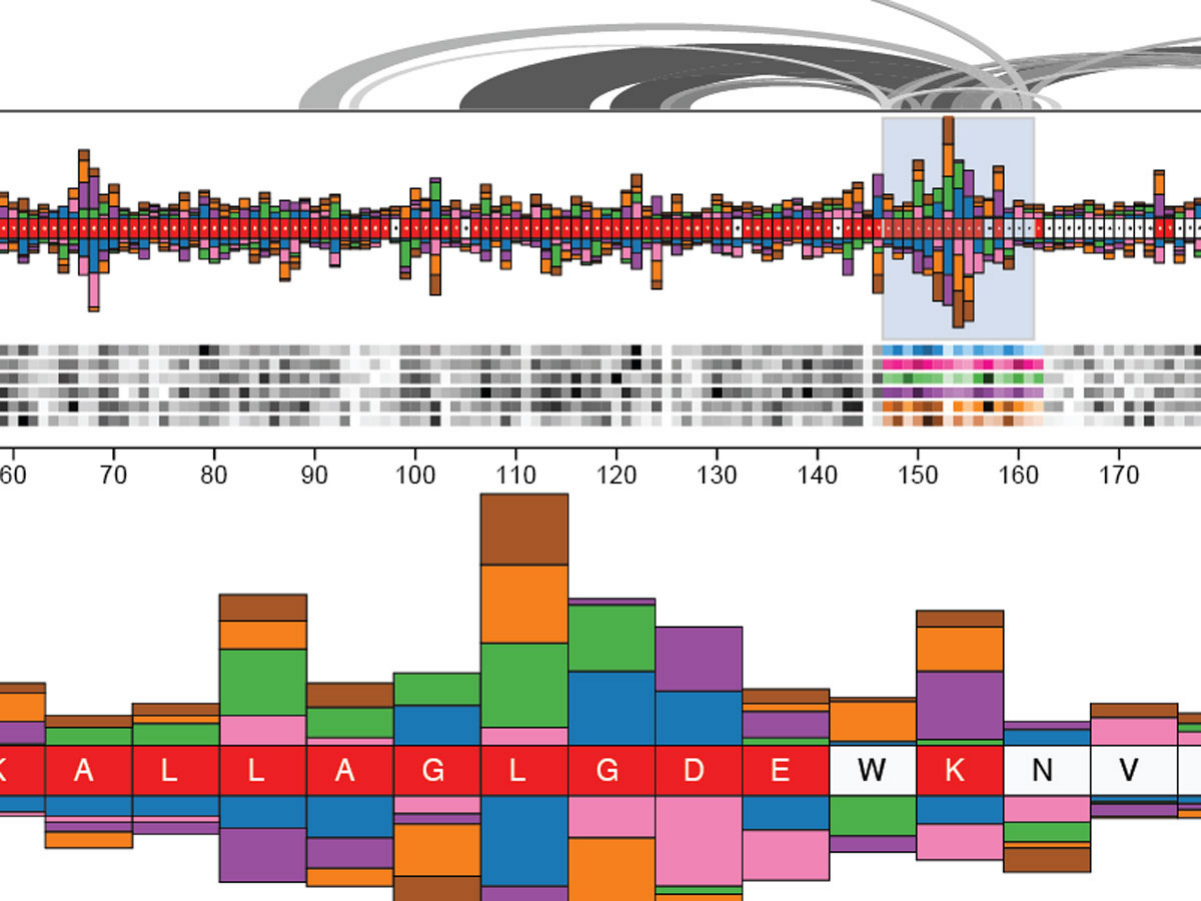
**Mu-8 in action**. Mu-8 provides a fluid and appealing interface for visualizing a selection of interesting features in TIM, and other regions of the protein to which this region is connected.

Mercer et al.[20] approached the problem by focusing their attention on the diversity of residues present at each position in the functional members of the protein family, and developed an interface around exploring this diversity. Their interface, Mu-8, centers around a bar-chart-like view of the sequence family and the mutant under consideration, where each sequential position displays a simple but powerful representation of the distribution of physicochemical properties used by the family at that position, and how the residue selected by the mutant fits these distributions. Around this chart, additional information is layered, such as 3D structure and arcs indicating which other residues in the sequence are in contact with any selected region.

Mu-8 has a very clean UI and presents easy to use visualizations of its analyses as shown in Figure 9. A significant strength derives from their application of a novel scoring algorithm that evaluates which physicochemical properties, or combination of properties, are most important in the family at each position. This enables the interface to evaluate "conservation" based on a different, contextually appropriate selection of properties, for each position independently. Coupled with a visualization of how diverse the functional sequence family is at each position based on this context-dependent score, and indication of where in this diversity the mutant fits (or doesn't fit), Mu-8 provides a simple way to identify where the mutant isn't like the family, without requiring the user to choose a meaning for "different" before performing the comparison.

Roca

**Figure 10:**
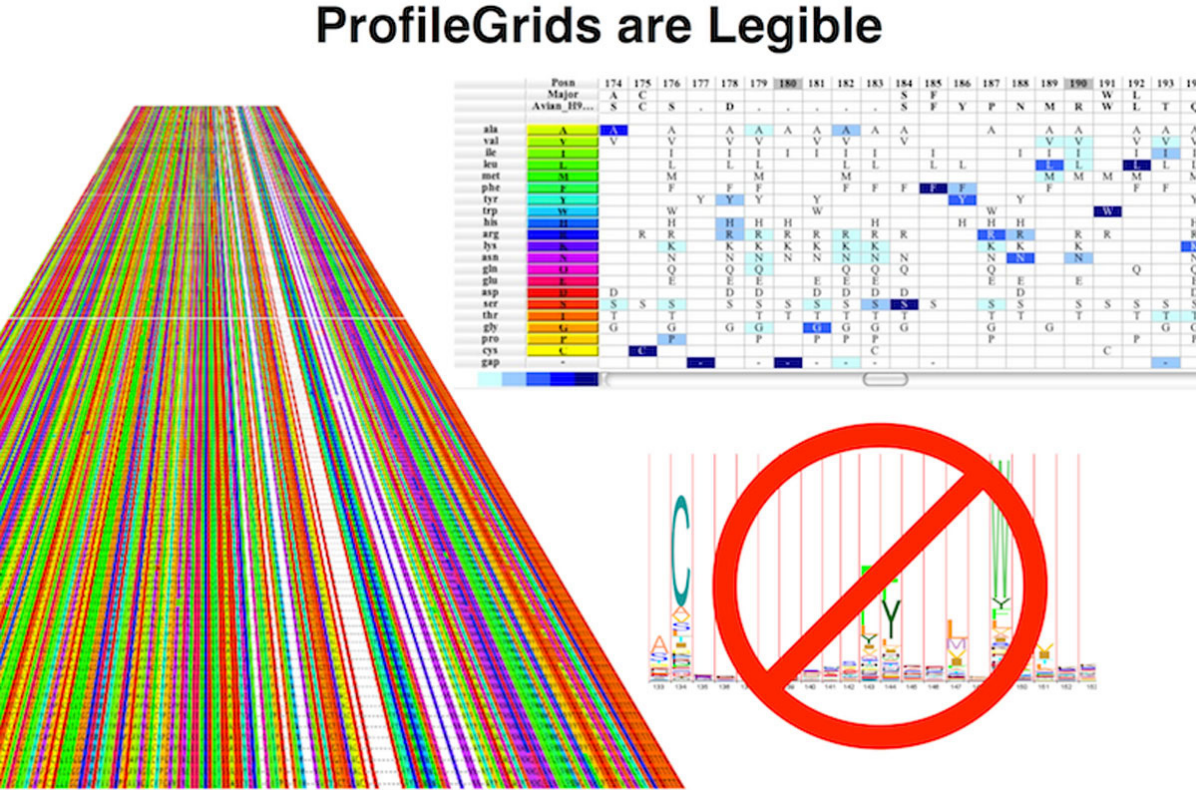
**ProfileGrids representation of positional frequency data**. ProfileGrids display simplified representations of massive tabular data and the conservation of different features in the tabular rows.

Roca[21] applied what may be the most deceptively simple technique, his categorical-data visualization approach (ProfileGrids shown in Figure 10), to the Data Analysis challenge. ProfileGrids are, most simply put, sensory recapitulations of the ubiquitous Position Specific Scoring (or position specific weight) Matrix[22]. Using a heat-map-like approach, ProfileGrids arrange sequential information (positions along the protein sequence) along the × axis, and categorical values (amino acids) along the Y axis in fixed locations, and then apply weighted shading to each resulting coordinate to indicate how frequently the family uses that particular residue, in that location.

While quite simple sounding, this approach conveys sequence diversity and conservation in an easy to use, simply accessible format, without requiring users to learn a new symbol language or way of reading information. By incorporating features that enable the user to group, sort and color amino acids by physical properties such as hydrophobicity, Roca's JProfileGrid tool enables facile examination of the sequence family residue distribution. By appropriate coloring and layering of outlines and other glyphs, a mutant sequence, or alternative families of sequences can be visually compared.

Knisley and Knisley

**Figure 11:**
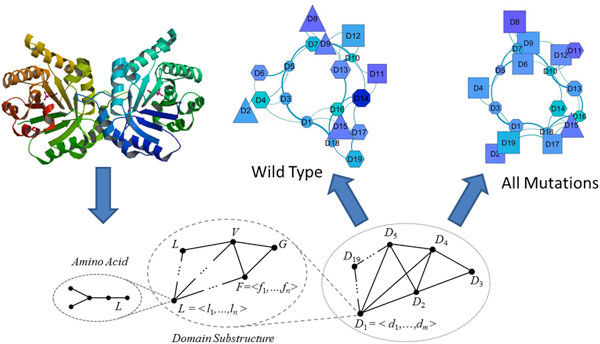
**Knisley's graph-theoretic approach**. Knisley's approach portrays the protein data as a hierarchy of network-connected graphs, from the atomic level, up through coordinated subsets of amino acids and groupings of these such as secondary or tertiary structures and domains.

Knisley and Knisley[23] presented the most theoretical approach to the problem, developing a multi-layer nested graph-theoretic model. Their approach casts the protein into a hierarchical graph structure with an overall protein view (domains as vertices), a domain view (residues as vertices), and an amino acid view (atoms as vertices), each with edges indicating physical or proximity-based interaction inferences between the features, as shown in Figure 11.

Each visualized graph conveys a different perspective of the effects of the mutation; for example, the top level graph of the domains very clearly shows how mutations cluster or distribute across protein domains. Applying a more formally theoretic approach, including elements derived from formal Chemical Graph Theory[24], Knisley's visualizations are rooted in formal theoretical graph structures rather than in ad-hoc presentations of the basic protein sequence or structure data. As a result their visualizations have more constrained representations and are somewhat less immediately accessible. Nonetheless their formal-theory based approach enables the discovery of fundamentally interesting properties of the proteins under analysis. For example, by filtering the top-level graph for certain types of biophysical features (such as hydropathy) and then filtering based on the domain localization of mutations, it's possible see that mutations in certain domains have little effect on contacts between hydrophilic residues, while mutations in other domains rearrange hydrophilic contacts significantly. Such intuition could be critical to understanding the biophysical basis of mutational effects, making the Knisley approach quite interesting, regardless of learning curve.

In addition to the interesting visualization features this nested-graph approach provides, casting the data into formal graph structures of this type enables the application of a voluminous body of rigorous and formal work from graph theory, to the usually ad-hoc task of comparing and contrasting the consequences of changes in the mutant, versus the protein-family uses of these residues.

### Redesign

**Winning Entry : Hofmann : **Hofmann was unable to submit a manuscript for these proceedings. Nonetheless Hofmann's approach to the Redesign contest was simple and elegant. Rather than stacking all residues present at a position on top of one another, Hofmann displayed the overall consensus residues above the baseline, and alternative residues below the baseline. This made for a very readable view - a user can easily identify the consensus residue because they all sit on the same baseline, and its relative occurrence frequency and positional information content, something often challenging in traditional Sequence Logos. This simple but insightful change makes the Hofmann approach to representing the features of a single sequence family much more user-friendly.

An additional view modification brought the ability to compare two sequence families more easily. To accomplish this, Hofmann interleaved the residue-propensity stacks for the two different families, within the same figure. In Hofmann's new comparative view, each position in the sequence is represented by a pair of residue-propensity stacks, one for each subfamily. This enables the user to easily see the differences in consensus between two classes (e.g. gram-negative and gram-positive). Both of these changes are simple, and render Hofmann's modified Sequence Logo more usable for the ADK lid data, allowing it to present more information with a less cluttered display.

**Tied for 2nd : Kultys et al**.

**Figure 12:**
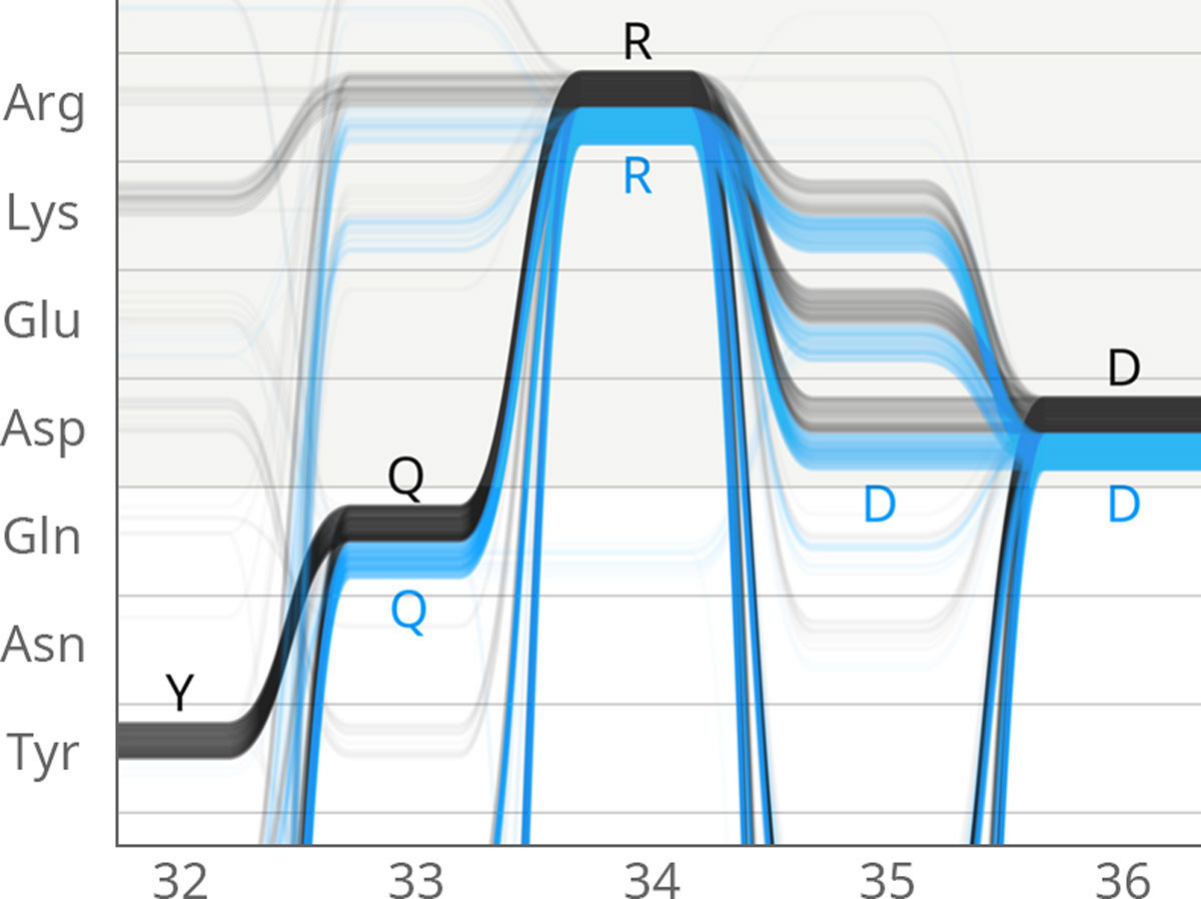
**Sequence Bundles showing differential AA choices**. Sequence Bundles use a representation similar to parallel coordinates to show the choices made by each member of the ADK family, highlighting the different choices made in by the gram-negative and gram-positive bacterial families of Adenylate Kinase.

Kultys et al.[17] developed a "Sequence Bundles" visualization to represent residue alignments. Related to parallel-coordinate[25] views, Sequence Bundles consists of multiple lines that traverse the sequential residue positions on the × axis, and move vertically to show residue identity at each position. Trends in residue usage frequency can be identified based on the weight of the line at a given position. At all locations the researcher can glean the presence and approximate distribution of any residue, as well as the relative distributions between the different ADK lid subfamilies. The order of the Y axis can be sorted in a variety of useful ways; for example grouping by chemical characteristics to see if variations have an impact on the nature of residue at a given location.

The Sequence Bundles method is an attractive representation which makes regions of strong conservation by identity, or by property - or broad distribution across many possible residue choices - easy to see, and easy to understand for the domain-scientist user. The Bundles tend to draw the eye towards regions of conservation and variability in an aesthetically pleasing manner. The display is exceptionally well suited for large collections of sequences, but also works for a pairwise comparison, allowing the researcher to see similarities and differences in the sequences clearly.

In concert with the tied entry from Sakai et al., Kultys' Sequence Bundles are the only entries that convey an easy understanding of dependencies between some positions. While limited to identifying dependencies between sequential neighbors in the sequence, this feature alone makes it possible to detect sequence features such as positions 19 and 20, where Lysine, Lysine (KK) appear as the subfamily consensus for the gram-positive(Figure 3:C) bacteria, but where vanishingly few of the members actually have Lysine in both positions.

Tied for 2nd: Sakai and Aerts, also entered in data analysis contest

**Figure 13:**
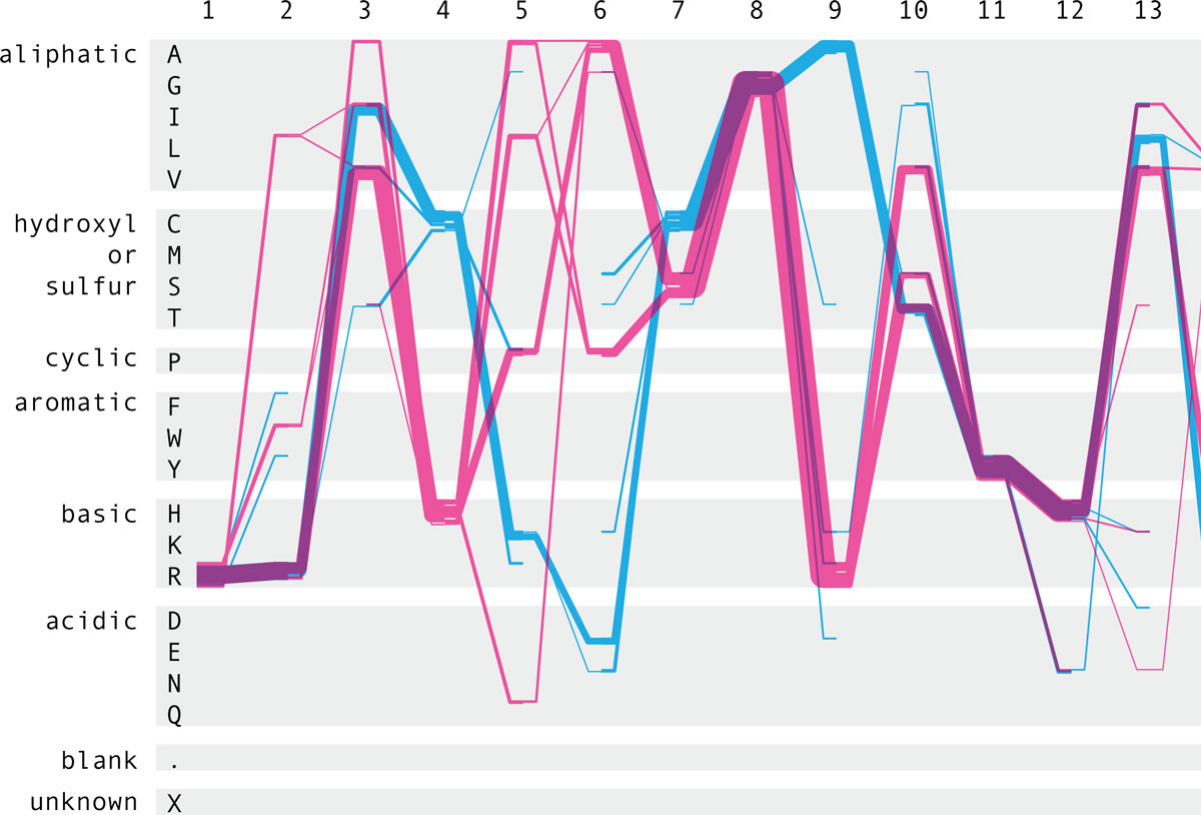
**A Sequence Diversity diagram**. Sequence Diversity Diagrams also show differential AA choices, and physicochemical property choices as used by gram-negative and gram-positive bacteria.

Sakai and Aerts[18] also improve on the Sequence Logo by creating a two-dimensional diagram called the Sequence Diversity Diagram, where the residues positions are on the × axis and the residue identities and physicochemical groupings are on the Y axis. As with the Sequence Bundles (above), a line is drawn through the corresponding residues for each position. Again, the line weights varies depending on the relative frequency of residues at consecutive positions.

The Sequence Diversity Diagram, like the Sequence Bundle, is a effective replacement for the Sequence Logo, and aids significantly in understanding the actual diversity and conservation of the sequence families, as well as how well an individual sequence follows the patterns of the family.

## Conclusion

The 2013 BioVis contests produced interesting and valuable contributions to both mutation assessment and presentation of family differences in proteins.

Judges of the Data Analysis contest found important insights and novel contributions in each of the entries, with several identifying components of known rescue mutants for dTIM, and all suggesting regions of dTIM that the domain experts felt were sufficiently interesting to warrant further study. Several of these new approaches and tools are already deployed and available for research use, as documented in the project-focused manuscripts in this proceedings. Demonstrating the need, and opportunity for continuing work in this domain, none of the approaches provide a complete view of the residue dependency networks present in TIM, the ways that dTIM violates these, or the several different ways that dTIM's functionality can be rescued. Nonetheless, in the consensus opinion of the judges, all of the approaches provide fundamental advances over the current state of the art for predicting the consequence of protein mutations, and all are worthy of consideration in protein engineering situations where a residue's contribution to protein function may not be independent of its neighbors.

Judges of the Redesign contest found considerable variation across the Redesign submissions, from entries that, in principle made worse use of visual salience, but did so in ways that were more familiar to typical users, to novel visualizations that would require re-training users in a new sequence-reading paradigm, but that conveyed some of the dependency-network characteristics necessary to understand the ADK lid families. Highlighting the need for continued improvement in sequence-family-comparison visualization, none of the entries completely convey the signature dependency networks, and none completely do away with inattention-blindness problems that occur when the user is forced to visually switch contexts between examining one family, or one feature, and another.

Despite the considerable advances over the state of the art produced by the Bio- Vis contest entries, it is also clear that there still remains much work in this field to be done. The original bioinformatic analysis that identified a working set of rescue mutations for dTIM[1], required months of hand analysis and painstaking mapping of conditionally-present (based on residue identity) residue interaction networks. While many of the contest entries found some components of these networks, or pointed to other residues that are biologically interesting or expected to be relevant in the appropriate sequence context, none of the entries identified a majority of the known rescue mutations. Much progress has been made in supporting and simplifying the tasks of the original hand analysis, but much work also remains to be done. The approaches presented in these proceedings, in demonstrating that traditional simple consensus approaches are *not *adequate, and that improved approaches that incorporate richer features and sources of information generate significantly improved results, have opened the door for continuing improvements in this field in the future.

## List of abbreviations

DNA: DeoxyriboNucleic Acid, AA: Amino Acid, ADK: denylate Kinase, TIM: Triosephosphate Isomerase, dTIM: defective TIM, scTIM: *Saccharomyces cerevisiae*, Mb: Megabytes, kD: kiloDalton.

## Competing interests

The authors declare that they have no competing interests.

## Authors' contributions

WCR, CWB and RM developed and ran the Data Analysis contest. MK and BW developed and ran the Redesign contest. TM, NC and BS developed the triosephosphate isomerase and adenylate kinase data sets, and answered domain-expert questions for contestants. RWR and WCR drafted and edited the manuscript. BS, NC, TM, MK, BW and CWB evaluated contest entries, contributed to contest entry descriptions, and assisted with manuscript preparation.

## Declarations

Publication of this work was supported by funds from the Research Institute at Nationwide Children's Hospital.

This article has been published as part of *BMC Proceedings *Volume 8 Supplement 2, 2014: Proceedings of the 3rd Annual Symposium on Biological Data Visualization: Data Analysis and Redesign Contests. The full contents of the supplement are available online at http://www.biomedcentral.com/bmcproc/supplements/8/S2
